# New Insights into Cranberry Bioactivity: Polyphenol Composition, Adhesive Effects Against Food Spoilage Yeasts, and Influence on Intestinal Cells

**DOI:** 10.3390/molecules31030418

**Published:** 2026-01-26

**Authors:** Dorota Kręgiel, Joanna Oracz, Karolina Czarnecka-Chrebelska, Adriana Nowak

**Affiliations:** 1 Department of Environmental Biotechnology, Faculty of Biotechnology and Food Science, Lodz University of Technology, Wolczanska 171/173, 90-530 Lodz, Poland; adriana.nowak@p.lodz.pl; 2 Institute of Food Technology and Analysis, Faculty of Biotechnology and Food Sciences, Lodz University of Technology, Bohdana Stefanowskiego 2/22, 90-537 Lodz, Poland; joanna.oracz@p.lodz.pl; 3 Department of Biomedicine and Genetics, Medical University of Lodz, Mazowiecka 5, 92-215 Lodz, Poland; karolina.czarnecka@umed.lodz.pl

**Keywords:** cranberry juice, polyphenols, adhesion, food spoilage yeasts, intestinal cells

## Abstract

The purpose of this study was to characterise the effect of cranberry (*Vaccinium macrocarpon*) juice on unicellular and multicellular systems, specifically food spoilage yeasts (*Wickerhamomyces anomalus*, *Dekkera bruxellensis* and *Rhodotorula mucilaginosa*) and intestinal cells (IEC-6 and Caco-2 cells). The effects of both raw cranberry juice and juice digested in vitro were investigated. The juices were analysed for polyphenol content using high-performance liquid chromatography coupled with mass spectrometry. The cranberry juice was evaluated for its impact on yeast surface hydrophobicity and anti-adhesive action using the MATH test and luminometry/microscopy, respectively. We also assessed the effects of raw and digested cranberry juices on IEC-6 and Caco-2 cells by measuring cell viability, metabolic modulation, genotoxicity, and antioxidant activity. Chromatographic analysis of the raw cranberry juice revealed the presence of diverse bioactive compounds, identified as hydroxybenzoic and hydroxycinnamic acids, flavonols, and anthocyanins. After digestion, the cranberry juice remained a rich source of phenolic acids. The yeast strain *R. mucilaginosa* was characterised by the highest hydrophobicity and adhesive abilities, but cell adhesion in the presence of raw cranberry juice was several times lower for all the tested strains. Both tested cranberry juices reduced ROS levels and were well tolerated by intestinal epithelial cells, without significant cytotoxic or genotoxic effects. Our findings provide new insights into the safety of using cranberry juice across unicellular and multicellular systems. However, further validation in real-world settings is necessary before practical applications.

## 1. Introduction

Cell adhesion is a key element for maintaining the collective functions of living organisms in nature. It is critical for the development and maintenance of both unicellular and multicellular organisms. Cellular adhesion and biofilm formation can have beneficial effects on human health, for example, by supporting stable microbial communities and maintaining barrier integrity. However, when these processes involve pathogenic organisms or become dysregulated, they may lead to infections and contribute to various diseases. Precise spatial and temporal regulation of cell adhesion is also essential during embryogenesis of multicellular organisms, including for gastrulation, tissue patterning, and organogenesis. Disruptions in cell adhesion can cause developmental abnormalities and congenital disorders. In adults, cell adhesion remains crucial for tissue homeostasis and function. Dysregulation of cell adhesion is linked to various pathological conditions, including cancer, autoimmune diseases, and tissue fibrosis [1]. In industrial settings, microbial adhesion and biofilm development are major sources of contamination and reduced process efficiency [2]. Therefore, given the importance of biofilms in healthcare and industry, developing a deeper understanding of cell adhesion processes is vital [3]. Taking the above into account, when examining the impact of specific substances with potential applications, it is important to consider multiple aspects, including both antimicrobial and anti-adhesive effects, as well as possible side effects and the influence of these chemicals on human health.

Natural plant extracts are increasingly the subject of anti-adhesion research. Cranberries, bearberries, pomegranates, green tea, and other phytochemicals with proven biological properties have attracted particular attention [4,5,6]. Plant-based strategies are generally well tolerated and can complement conventional therapies. For example, cranberry extracts have become an element of innovative antibiofilm strategies [7,8,9]. Years of research have documented that cranberries are rich in bioactive substances, including phenolic compounds that are important for maintaining human health [10].

The mechanisms of cranberry actions were extensively examined by Ottaviano et al. [11] who identified urinary metabolites from cranberries that could enable drug development by targeting the very early stages of biofilm formation and preventing genitourinary *Candida* infections. Recent research has shown that the combination of cranberry juice and lactic acid bacteria significantly inhibits *C. albicans* adhesion and prevents vulvovaginal candidiasis [12]. In vitro studies have described the cytoprotective effect of a cranberry fraction rich in proanthocyanidins on cell damage and death in Madin-Darby canine kidney (MDCK) cells [13], as well as the inhibition of proliferation in human tumour cell lines [14], selective targeting of acute myeloid leukaemia cells [15], and inhibition of oesophageal adenocarcinoma [16]. Modulation of reactive oxygen species in Barrett’s and oesophageal adenocarcinoma cells has also been reported, along with the suppression of lipopolysaccharide-stimulated macrophage and normal gingival fibroblast activities [17,18]. However, older adults in nursing homes, pregnant women, and patients with chronic illnesses may not gain benefits from consuming cranberry products. Cranberries should be avoided by those allergic to *Vaccinium* spp. Some adverse effects have been reported in patients taking cranberry alongside warfarin, a common anticoagulant used to prevent and treat venous thrombosis, thromboembolic events, myocardial infarction, and atrial fibrillation [19,20,21]. Therefore, studies on the effects of cranberries require further high-quality clinical trials to verify their efficacy, safety, and suitability for personalised treatment.

Despite extensive research on the inhibitory effects of cranberry polyphenols on common pathogens and their impact on various cytoprotective mechanisms, knowledge of the effects of cranberries on food spoilage microorganisms remains rather limited. Recently, concerns about hygiene, the environment, and health have drawn attention to cranberry compounds as an alternative to synthetic food preservatives and digestive aids. Ten years ago, preliminary studies investigated the effects of cranberry juice on Gram-negative bacteria of the genus *Asaia*, which cause specific contaminants in the beverage industry [22]. However, the behaviour of food spoilage yeasts in the presence of cranberry juice remains less studied compared to research on common pathogens. Furthermore, only a few studies on the effects of cranberries on the gastrointestinal tract have been conducted [23,24]. Our main aim was therefore to expand existing knowledge on the adhesive capacity of food spoilage yeasts in the presence of raw cranberry juice. We also investigated the cytotoxic, genotoxic, and antioxidant effects of raw and digested cranberry juice using both normal and cancerous intestinal cell models. This broadens the scope of comprehensive research on cranberry juice.

## 2. Results and Discussion

Plant polyphenols influence the adhesion of both microbial cells and various cell lines [25,26,27]. Cranberries and their polyphenols can also potentially help control infections by modulating biofilm formation [28]. Therefore, this study investigated the polyphenol profiles of cranberry juices.

### 2.1. Polyphenol Profile of Cranberry Juice

The phenolic acids and flavonoids in fresh cranberry pulp and prepared juice before (raw) and after simulated digestion were measured by liquid chromatography. The results are shown in Table 1, presented as the arithmetic means from three samples with the standard deviation. In the tested samples, a total of 39 phenolic compounds were identified, including (1) 14 hydroxycinnamic acids and their derivatives: gallic acid, 3,4-dihydroxybenzoic acid, 3,5-dihydroxybenzoic acid, protocatechualdehyde, 4-hydroxybenzoic acid, 2,5-dihydroxybenzoic acid, 2,4-dihydroxybenzoic acid, vanillic acid, 3-hydroxybenzoic acid, 2,3-dihydroxybenzoic acid, syringic acid, syringaldehyde, benzoic acid, 3-(2-hydroxyphenyl)propanoic acid; (2) 8 hydroxycinnamic acids and their derivatives: chlorogenic acid, neochlorogenic acid, *p*-coumaric acid and *p*-coumaric acid-*O*-hexoside, 4-*O*-caffeoylquinic acid, ferulic acid, sinapic acid, 3,4-di-*O*-caffeoylquinic acid; (3) 13 various flavonols based on myricetin, quercetin and their derivatives: myricetin 3-*O*-galactoside, myricetin 3-*O*-xyloside, myricetin 3-*O*-arabinoside, quercetin 3-*O*-glucosyl-rhamnosyl-glucoside, quercetin 3-*O*-galactoside, quercetin 3-*O*-rutinoside, quercetin 3-*O*-xyloside, quercetin 3-*O*-coumaroylgalactoside, quercetin 3-*O*-arabinopyranoside, quercetin-3-*O*-arabinouranoside, quercetin 3-*O*-rhamnoside; and (4) 4 anthocyanins: cyanidin 3-*O*-galactoside, cyanidin 3-*O*-arabinoside, peonidin 3-*O*-galactoside, peonidin 3-*O*-arabinoside.

The total content of phenolic compounds, calculated as the sum of all identified compounds in the cranberry juice, ranged from 48.33 to 66.44 mg/100 mL. In other studies, tested cranberry juices obtained in Poland contained polyphenols in the range of 80–185 mg/100 mL, expressed in gallic acid equivalents. The average polyphenol content before opening the juices remained constant. Of course, the season of harvest and the technology of juice production should also be taken into account in such analyses [29].

In the present study, the predominant phenolic compounds in the raw juice before digestion were anthocyanins (20.60 mg/mL) and flavonols (21.30 mg/100 mL). Hydroxybenzoic acids and hydroxycinnamic acids were present in slightly smaller amounts (respectively, 13.90 mg/100 mL and 10.63 mg/100 mL). These findings align with those of other researchers who have identified similar phenolic compounds in cranberry juice [30,31].

Our study showed that the profiles and concentrations of individual compounds in cranberry juice change significantly during simulated digestion. The total phenolic content dropped to 30.78 mg/100 mL, representing an over 50% decrease compared to the initial raw juice. During intestinal digestion, complex phenolic compounds including phenolic acid derivatives and flavonoids can break down into individual phenols, such as syringic acid, cinnamic acid, caffeic acid, and protocatechuic acid [32]. The most notable reduction was seen in the anthocyanin content, as none of the previously identified anthocyanins were detected in cranberry juice after digestion. It can be assumed therefore that the anthocyanin content of cranberry juice decreases markedly during simulated gastric digestion and is further reduced during the intestinal phase. These conclusions are consistent with the existing literature [33].

The structure of anthocyanins undergoes significant transformation during digestion, driven by pH fluctuations and the activity of intestinal microflora. Due to their high reactivity, anthocyanins are readily converted, leading to structural modifications. During digestion, anthocyanins can be converted into several different metabolites, including chalcone glycosides, phenolic acids, aldehydes, and other simple phenols [34]. The extent of these changes depends on the structure of the anthocyanins, including the type of aglycone, B-ring substitution, the nature and degree of esterification and glycosylation, and the number and positions of their oxygenated substituents. Anthocyanins exhibit high stability at the low pH of the stomach, but their chemical structures alter as pH increases [35,36]. In line with the reduction in anthocyanin content during digestion, a similar decline in flavonol levels was observed. The amount of myricetin, quercetin, and their derivatives in the juice after digestion was only 9.90 mg/100 mL. These results align with those of other researchers, who reported that although myricetin and quercetin remain stable under gastric conditions, they undergo accelerated degradation in the intestinal tract [37].

Our study found that phenolic acids and their derivatives were more stable during cranberry juice digestion. The contents of hydroxybenzoic acids and hydroxycinnamic acids in cranberry juice after digestion were 10.92 and 9.95 mg/100 mL, respectively. The observed increase in the concentration of total hydroxybenzoic acids and their derivatives, particularly gallic acid, dihydroxybenzoic acids, and syringic acid, as well as the appearance of two new compounds (benzoic acid and 3-(2-hydroxyphenyl)propanoic acid), is likely related to the degradation of flavonoids, mainly anthocyanins. This study also indicates that in vitro digestion of cranberry juice alters the composition of hydroxycinnamic acids. The most notable decrease was seen in 3,4-di-*O*-caffeoylquinic acid, which was completely lost after digestion. However, an opposite trend was observed for *p*-coumaric acid, ferulic acid, sinapic acid, and 3-*O*-caffeoylquinic acid. The increase in the concentration of these phenolic acids after in vitro digestion can be explained by the breakdown of more complex phenolic compounds, including hydroxycinnamic acid derivatives and certain flavonoids [34,37].

The results of this study demonstrate that the digestion of cranberry juice may cause significant structural changes in its phenolic compounds. However, even after passing through the digestive system, cranberry juice remains a source of active phenolic compounds, mainly phenolic acids.

### 2.2. Hydrophobicity of Yeast Cells

The strength of adhesion depends on a combination of physical forces (such as attractive van der Waals and electrostatic forces, including z-potential) and chemical forces (such as cohesive interactions between microbial cells) [38]. In microbial cells, adhesiveness influences surface hydrophobicity or hydrophilicity, as well as the capacity to secrete extracellular substances that facilitate contact [39]. In this context, cell surface hydrophobicity (CSH) seems to be critical for attachment to or detachment processes [40].

The topic of yeast cell hydrophobicity is intriguing, especially considering the potential capabilities of specific strains that are common contaminants in the food industry. Therefore, we explored the assessment of the CSH of food spoilage yeasts isolated from contaminated products. The literature broadly presents research on the hydrophobic properties of *Candida* spp. [41,42,43] and *Saccharomyces* spp. [44,45], as well as the effect of CSH on their flocculation abilities. We have extended this research to include other yeasts from the genera *Wickerhamomyces*, *Dekkera*, and *Rhodotorula* isolated from spoiled drinks in Poland. We recognise that the three selected yeast species present a certain methodological limitation, as many different yeasts can cause food contamination, and these should also be included in future research.

*W. anomalus* has been previously classified as *Hansenula anomala*, *Pichia anomala*, and *Candida pelliculosa*, which was recently reclassified into the genus *Wickerhamomyces* following phylogenetic analysis of its genetic sequence. *W. anomalus* is responsible for the spoilage of food products [46]. However, many studies suggest that this yeast has potential as a biocontrol agent [47,48]. It is worth noting that reports of human infections by *W. anomalus* have indicated that this yeast may also be an emerging pathogen [49]. In turn, *Dekkera*/*Brettanomyces bruxellensis* and *R. mucilaginosa* were found in several fermented matrices and cause significant alterations to the final product quality [50,51]. *R. mucilaginosa* is one of the most commonly encountered pathogens in infections associated with underlying immunosuppression or cancer [52]. The three yeast strains used in this study were isolated in Poland from spoiled soft drinks with characteristic flocs as a visual defect. They were able to form consortia with acetic acid bacteria *Asaia* spp. [53].

**Figure 1:**
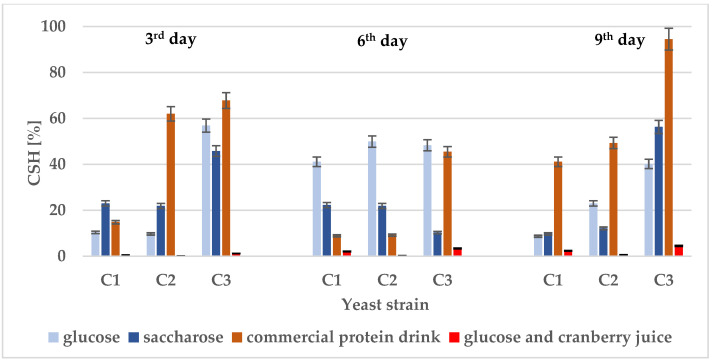
Cell surface hydrophobicity (CSH) of yeast strains: *W. anomalus* C1, *D. bruxellensis* C2, and *R. mucilaginosa* C3 in different environments: minimal medium with glucose, minimal medium with saccharose, a commercial protein drink, and minimal medium with glucose and 10% (*v*/*v*) cranberry juice on the 3rd, 6th, and 9th day of incubation.

Our previous studies demonstrated that yeast cell hydrophobicity may vary depending on the yeast species and the chemical composition of the culture medium. Then, *R. mucilaginosa* displayed the strongest hydrophobic properties, especially in enriched medium with glucose (75%) and commercial flavoured mineral water with saccharose (45%) [53]. This fact was also confirmed in the present study. The highest CSH results for *Rhodotorula* strain were recorded this time in a commercial protein drink (45–95%) (Figure 1). The results also confirmed that yeast hydrophobicity is dynamic over time and depends on the culture environment. It is worth noting that a significant decrease in CSH was observed for all tested strains in the culture media with cranberry juice. In the presence of 10% raw cranberry juice, CSH was from a dozen to several dozen times lower. A detailed statistical analysis of the results, including both horizontal comparisons of CSH between yeast strains in different environments and comparisons of hydrophobicity trends in different culture environments, is presented in the Supplementary Material (Tables S1 and S2).

Cell surface hydrophobicity, as a complex property of the cell surface, can be modified by various components of the cell wall. For example, mannoproteins, glucans, lipids, and chitin influence CSH in yeast *C. albicans* [54]. However, under different stress conditions, yeasts activate networks of signalling pathways that regulate the overall transcriptional response and initiate coordinated cellular changes. These pathways work together to regulate the composition, organisation, and biophysical properties of the cell wall. In the presence of acids, the cell wall structure and composition may change, altering the cell surface morphology. For example, the level of chitin in the cell wall decreases at below pH 5.0, likely due to increased chitinase activity. Acid conditions also affect the β-glucan fraction in the cell wall, which in turn influences the CSH of microbial cells [55].

Polyphenols can influence microbial CSH. According to the literature, they form complexes with polysaccharides through various noncovalent interactions—hydrogen bonds, hydrophobic forces, and electrostatic attractions [56]. Similar mechanisms can also be attributed to organic acids, which may inhibit biofilm formation. Therefore, it can be assumed that the combination of organic acids and polyphenols may exert a synergistic inhibitory effect on microbial biofilms [10,55,57].

We observed significantly different CSH values in the presence of cranberry juice. In such a complex, poorly defined environment, pH correction may affect hydrophobicity results, depending on the presence of various bioactive phenolic compounds. In studies conducted by Mekoue Nguela and co-workers, the whole cells exhibited a high capacity to adsorb grape wine tannins irreversibly [58]. Microscopy observations indicated that, if tannins interact with cell walls, especially cell wall mannoproteins, they may also diffuse freely through the walls of inactivated cells to interact with their plasma membrane and cytoplasmic components. In turn, Morey and co-workers evaluated the effects of tannin fractions from *Stryphnodendron adstringens* on the growth of *C. tropicalis* and its adhesive properties. Exposure to planktonic yeast cells significantly decreased cell surface hydrophobicity and cell adhesion. A strong fungistatic effect was also observed, resulting in a notable reduction in biofilm mass [59].

### 2.3. Adhesion of Yeast Strains

**Figure 2:**
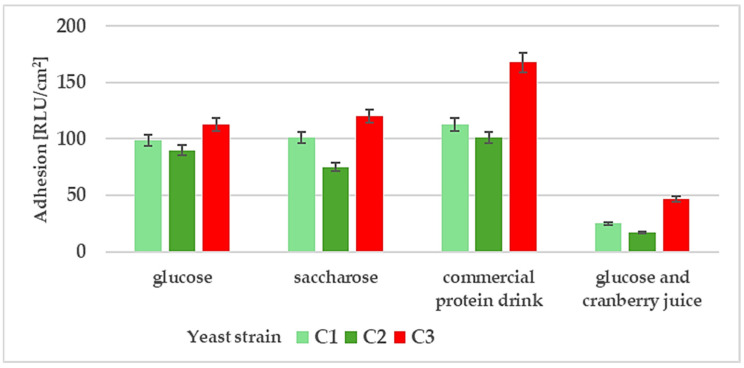
Adhesion of yeast cells: *W. anomalus* C1, *D. bruxellensis* C2, and *R. mucilaginosa* C3 in various environments: minimal medium with glucose, minimal medium with saccharose, commercial protein drink, and minimal medium with glucose and 10% (*v*/*v*) cranberry juice, after 9 days of incubation.

**Figure 3:**
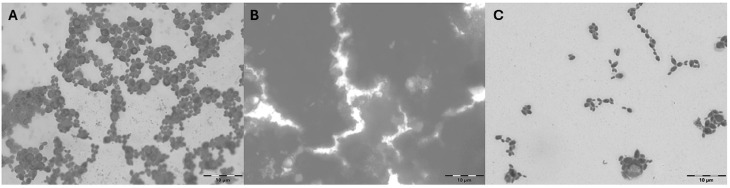
Adhesion of *R. mucilaginosa* C3 on the glass surface in: (**A**) minimal medium with glucose, (**B**) commercial protein drink, and (**C**) minimal medium with glucose and 10% (*v*/*v*) cranberry juice. Bars represent 10 μm.

To examine microbial cell adhesion, we used glass microscope slides as substrates. Such incubation conditions were used in our previous studies [22,53]. Yeast cells were cultivated in flasks, and cell staining followed by visualisation proved to be an effective method for studying adhesion. Microfouling on coated glass slides was also analysed using the adenosine-5′-triphosphate (ATP) method (luminometry). This technique is frequently utilised in adhesion research [53,60,61]. The results of cell adhesion after long-term incubation (9 days) in different culture media are shown in Figure 2 and Figure 3.

The strain *R. mucilaginosa* C3 showed the best adhesion to the glass substrate. The results for this strain were nearly twice as high as those for the other strains in a medium containing a commercial protein soft drink (Figure 2 and Figure 3, Tables S3 and S4). Adhesion analysis revealed statistically lower adhesion of *D. bruxellensis* compared to *R. mucilaginosa*. It can be assumed that the high CSH content of this strain also contributed to its strong adhesion in this medium. In addition, we confirmed that environmental proteins (in this case, a commercial protein drink) may improve adhesion even on hydrophilic substrates [62]. In contrast, there were significant differences in adhesion in minimal culture medium supplemented with cranberry juice across all tested yeast strains (Tables S3–S5). For example, the adhesion of *R. mucilaginosa* C3 was twice as low in the presence of cranberry juice compared to the media without cranberry supplementation. Our results for food spoilage yeasts support previous reports on the role of cranberry juice in reducing bacterial adhesion, notably for *E. coli* [63], *Salmonella* Typhimurium [64], and *Asaia* spp. [22]. For example, for the strain of acetic acid bacteria *A. bogorensis* characterised by strong adhesion properties, cell attachment was three times lower in the presence of 10% cranberry juice [22].

The mechanisms by which cranberry juice components inhibit yeast adhesion vary. Feldman and colleagues suggested that A-type cranberry proanthocyanidins from cranberry extracts are responsible for reducing the adhesion of *C. albicans* to oral epithelial cells [65]. However, despite promising in vitro results, it is important to note that laboratory studies should be continued to verify the results using different surfaces, yeast strains, and, most importantly, controls under industrial conditions, with particular attention to validation requirements. The real secondary food contamination caused by biofilms are the result of the long-term existence of biofilms on production surfaces. These facts could offer a new perspective on cranberries as a component that reduces contamination risk in the food industry and as a bioactive agent with significant therapeutic potential.

### 2.4. Viability and Metabolic Modulation of Intestinal Epithelial Cells

**Figure 4:**
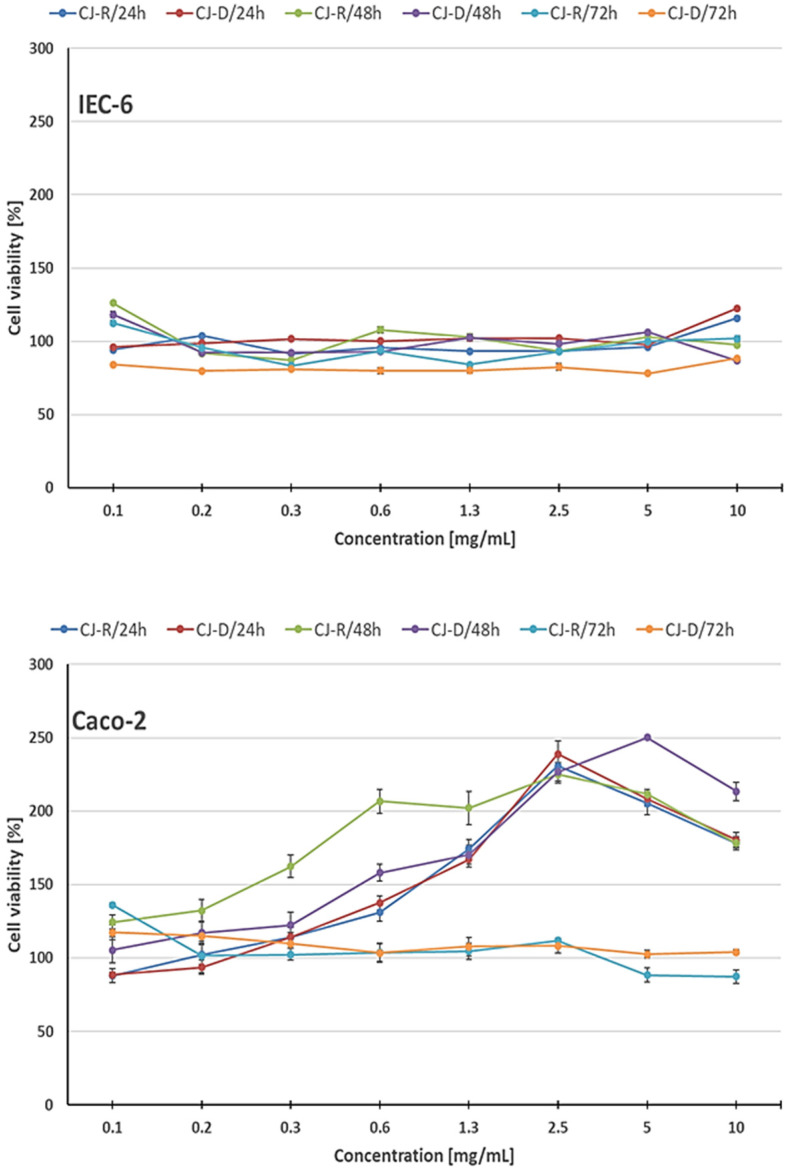
Effect of raw (R) and digested (D) cranberry juice (CJ) on the viability of Caco-2 and IEC-6 cells in MTT assay after 24, 48 and 72 h exposure. Each value represents the mean of four repetitions ± the standard deviation (SD).

In IEC-6 cells, viability remained close to control levels across the entire concentration range (0.1–10 mg/mL) and at all time points (24, 48, 72 h) (Figure 4 and Figure S1). Instead of a clear dose–response relationship, the curves showed slight, unsystematic fluctuations. A modest increase in viability (approximately 110–130% of the control) was noted at some low and medium concentrations and at specific time points (especially after 48 h), while higher concentrations generally returned to baseline levels. Overall, neither raw nor digested cranberry juice caused significant (*p* ≤ 0.05) cytotoxicity in IEC-6 cells. Digested juice showed a slightly weaker effect than raw juice, but the differences were slight and inconsistent across concentrations and time points.

Exposure of Caco-2 cells to both raw and digested cranberry juices did not cause cytotoxicity at any of the tested concentrations or exposure durations (Figure 4 and Figure S1). After 24 and 48 h of incubation, a concentration-dependent increase in cell metabolic activity was observed, particularly at lower and medium concentrations of both types of juice. It should be noted that the MTT assay measures mitochondrial dehydrogenase activity and reflects cellular metabolic activity rather than overall cell health or function. The moderate increases observed indicate a temporary stimulation of mitochondrial activity, which returned to baseline at higher concentrations or after prolonged exposure, without corresponding cytotoxic effects. After 24 h, cell viability increased with concentration, reaching approximately 240% of the control value at the highest concentrations (2.5–5.0 mg/mL) (*p* ≤ 0.05). Similar effects were noted after 48 h, with viability remaining elevated compared to the control, and raw juice inducing slightly stronger stimulation than digested juice. After 72 h of exposure, the metabolic activity of Caco-2 cells approached control levels, indicating that the initial stimulatory effect was transient. No statistically significant (*p* ≤ 0.05) cytotoxicity (viability <80%) was observed under any of the tested conditions.

Despite the lower pH of raw cranberry juice compared to digested cranberry juice, no increased cytotoxicity was observed in IEC-6 or Caco-2 cells. This indicates that the moderate acidity of the juice did not adversely affect cell viability, likely due to the culture medium’s buffering capacity and internal mechanisms that regulate cell pH. Overall, both raw and digested cranberry juices were well tolerated by Caco-2 cells, and short-term exposure resulted in a moderate increase in mitochondrial activity rather than inhibition. The observed moderate increases in cellular metabolic activity at low and medium concentrations of both raw and digested cranberry juice indicate a hormetic effect, characterised by a biphasic dose–response relationship. This transient stimulation, followed by a return to baseline at higher concentrations or longer incubation times, suggests that cranberry components may temporarily increase mitochondrial activity without inducing cytotoxicity.

Our findings regarding the negligible cytotoxicity of both raw and digested cranberry juice on Caco-2 cells align with previous reports indicating that cranberry polyphenols are well tolerated by intestinal epithelial models. González de Llano et al. [66] observed that Caco-2 cells maintained high viability after exposure to cranberry polyphenol preparations, supporting the view that these bioactive compounds may exert beneficial effects without compromising intestinal cell integrity. Cranberry pomace extract did not adversely affect the viability of A-549 lung epithelial cells at concentrations up to 400 µg/mL [67], which is consistent with our findings regarding the negligible cytotoxicity of whole cranberry juice on intestinal epithelial cells at concentrations up to 10 mg/mL. Although the extract type and cell models differ, this provides further evidence for the biocompatibility of cranberry-derived products. Weh et al. [68] reviewed several studies showing that cranberries can reduce viability and induce apoptosis in cancer cell lines. However, the authors note a relative lack of data on the cytotoxic effects of whole cranberry juice or digested preparations on non-cancerous intestinal epithelial models. Our results fill this gap by showing that even at relatively high concentrations (up to 10 mg/mL), raw and digested cranberry juices do not significantly reduce the viability of IEC-6 and Caco-2 cells. Our study thus confirms the biocompatibility of these preparations under conditions relevant to their use as food preservatives.

### 2.5. Genotoxic Effects

**Figure 5:**
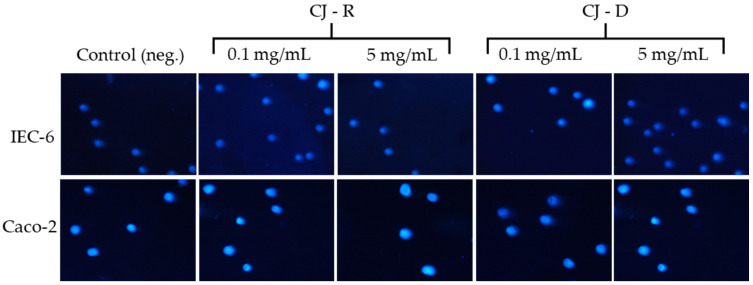
Examples of microphotographs of comets induced by raw (R) and digested (D) cranberry juice (CJ) after DAPI staining, analysed under a fluorescent microscope (Nikon Eclipse Ci H600L, Tokyo, Japan) with 20× objective.

The genotoxicity of both raw and digested cranberry juice was evaluated in IEC-6 and Caco-2 cells at concentrations ranging from 0.1 to 5 mg/mL (Table 2, Figure 5). These concentrations were chosen based on preliminary cytotoxicity data indicating that the juices are well tolerated within this range, as well as based on physiologically relevant levels of cranberry polyphenols achievable in the intestinal lumen after dietary intake.

The percentage of DNA damage in the comet tail in the negative control for IEC-6 cells was 5.63% ± 0.51%, while for Caco-2 cells it was 3.37% ± 0.78%. In IEC-6 cells, the percentage of DNA in comet tails remained low (1.56–7.19%), with no clear dose–response pattern and no consistent differences between raw and digested juice. Likewise, Caco-2 cells exhibited minimal DNA damage, with the highest values recorded at 5 mg/mL (8.72 ± 2.36% for raw juice and 7.38 ± 1.24% for digested juice). These findings indicate that neither raw nor digested cranberry juice caused significant genotoxic effects in intestinal epithelial cells under the conditions tested. The absence of a clear dose-dependent effect and the generally low level of DNA damage suggest that cranberry constituents are unlikely to compromise genomic integrity at levels associated with dietary exposure.

According to the literature, cranberry juices and extracts do not exhibit genotoxic effects. Instead, antigenotoxic (protective) effects are often observed. A study by Madrigal-Santillán et al. [69] showed that cranberry extract prevented DNA damage caused by benzo[a]pyrene in mice, without showing genotoxicity. This supports our findings of minimal DNA damage in intestinal epithelial models after exposure to cranberry juice. However, our study involves human and rat intestinal cells in vitro and tests whole juice (raw and digested) rather than an isolated extract. According to the EFSA [70], cranberry extract powder is deemed safe for the proposed conditions of use, with no evidence of cytotoxicity or genotoxicity. Their assessment included both in vitro tests and toxicological data, which did not indicate any mutagenic risk or other adverse effects. These results align with our observations, which show that both raw and digested cranberry juice had no cytotoxic or genotoxic effects at the concentrations used. Overall, both raw and digested cranberry juices were well tolerated by intestinal epithelial cells, with no significant cytotoxic or genotoxic effects observed across the entire concentration range tested. These findings suggest that cranberry juice is safe for intestinal cells, even after simulated gastrointestinal digestion, and supports its potential use as a natural food preservative.

### 2.6. Antioxidant Activity

**Figure 6:**
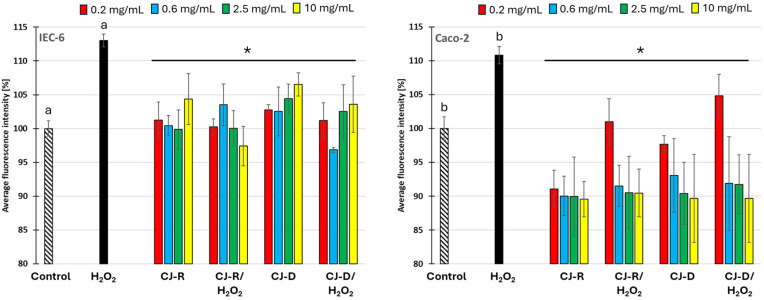
Effect of raw (R) and digested (D) cranberry juice (CJ) on ROS generation in Caco-2 and IEC-6 cells after 2 h of exposure, either alone or in combination with 200 µM H_2_O_2_. Data are presented as the mean ± SD of four replicates. Asterisks (*) denote significant differences relative to the positive control (200 µM H_2_O_2_); the same letters indicate statistical differences (ANOVA, *p* ≤ 0.05).

The antioxidant potential of raw and digested cranberry juice was additionally assessed by measuring the level of intracellular reactive oxygen species (ROS) in IEC-6 cells (Figure 6). Exposure to hydrogen peroxide (H_2_O_2_, 200 µM) increased ROS production to approximately 113% of the control value, confirming a significant (*p* ≤ 0.05) induction of oxidative stress. Treatment with cranberry juices alone did not significantly alter ROS production at the tested concentrations (0.2–10 mg/mL), indicating no pro-oxidative effect. When cells were co-treated with H_2_O_2_ and cranberry juice, ROS levels decreased to levels comparable to those in the untreated control (*p* ≤ 0.05), indicating a protective antioxidant effect.

In Caco-2 cells, treatment with 200 µM H_2_O_2_ increased intracellular ROS production to approximately 111% of the control value (*p* ≤ 0.05), confirming the induction of oxidative stress (Figure 6). Both raw and digested cranberry juices, used at concentrations ranging from 0.2 to 10 mg/mL, significantly reduced ROS levels (*p* ≤ 0.05), even below the level of untreated cells, demonstrating a clear antioxidant effect. When co-administered with H_2_O_2_, cranberry juices effectively alleviated oxidative stress (*p* ≤ 0.05), restoring ROS values to levels similar to those in the control. No clear dose–response relationship was observed in either cell line; cranberry juice reduced ROS levels at all tested concentrations without a consistent trend.

Both intestinal cell lines showed a consistent antioxidant response to exposure to cranberry juice. In IEC-6 cells, treatment with raw or digested juice alone did not alter ROS levels compared to the control, and co-treatment with H_2_O_2_ effectively counteracted the induced oxidative stress (*p* ≤ 0.05). Similarly, in Caco-2 cells both types of juice significantly (*p* ≤ 0.05) reduced intracellular ROS, even below control levels, and prevented ROS accumulation after exposure to H_2_O_2_. The effects were comparable for different concentrations (0.2–10 mg/mL) and between raw and digested samples, indicating that digestion did not reduce the antioxidant potential of cranberry juice. This effect was particularly visible in the Caco-2 line, where most concentrations, especially the lower doses, reduced ROS below control values. Overall, the data show that cranberry juice exhibits protective effects against oxidative stress in both normal (IEC-6) and human intestinal barrier (Caco-2) models, confirming its biocompatibility and potential as a natural preservative with antioxidant properties.

It is worth noting that in many cases, digested cranberry juice showed activity comparable to or even stronger than raw juice, suggesting that the digestion process did not reduce the antioxidant properties, and that some metabolites released during digestion may have further increased the bioavailability and effectiveness of phenolic compounds. Our results are consistent with many reports indicating that cranberry polyphenols may act as effective free radical scavengers or modulators of redox pathways. Some studies have shown that a comparable reduction in ROS levels (10–30%) was observed with cranberry extracts in intestinal epithelial cells, liver cells, fibroblasts, and macrophages, mainly through direct neutralisation of radicals and activation of endogenous antioxidant systems [71,72,73,74]. The absence of a clear dose–response relationship in our study suggests a hormetic response, where low concentrations of polyphenols are enough to offer maximum antioxidant protection [75].

Our findings align with recent original research demonstrating that metabolites derived from flavonols and anthocyanins exert cytoprotective effects in intestinal epithelial cell models. These effects mainly occur through free radical scavenging, modulation of redox-sensitive signalling pathways, and protection against oxidative DNA damage. Polyphenol-rich plant extracts and their digestion-derived metabolites have been shown to reduce intracellular ROS levels by around 20–40% in intestinal epithelial cells exposed to oxidative stress, most often caused by H_2_O_2_. These antioxidant effects are typically accompanied by a significant decrease in oxidative DNA damage, with comet tail DNA values often falling by 30–60% compared to oxidant-treated controls. Such cytoprotective responses usually happen at relatively low polyphenol concentrations, supporting our observation of effective ROS reduction without a clear dose–response relationship [76]. Consistent with our findings on preserved genomic integrity, it has been reported that polyphenol-rich plant extracts at non-cytotoxic and non-genotoxic concentrations (0.04% and 0.08%) significantly promote DNA repair in Caco-2 cells after oxidative stress induced by H_2_O_2_, suggesting that polyphenol-mediated cytoprotection may involve activating cellular DNA repair mechanisms [77]. Similar evidence from other phenol-rich plant juices further supports this idea. For example, phenolic compounds from Viburnum opulus juice decreased intracellular ROS production by approximately 15–25% and improved DNA repair in Saos-2 cells at non-cytotoxic concentrations of 50–200 µg/mL [78]. Likewise, in Caco-2 cells exposed to oxidative stress caused by tert-butyl hydroperoxide (t-BOOH) or H_2_O_2_, phenol-rich Viburnum opulus extracts at non-cytotoxic concentrations (IC_0_ around 50 µg/mL) significantly lowered intracellular ROS levels and enhanced DNA repair, demonstrating that complex phenolic mixtures can support genome integrity under oxidative stress conditions [79].

The combined assessment of cytotoxicity, genotoxicity, and antioxidant activity indicates that both raw and digested cranberry juice exert a marked cytoprotective effect on intestinal epithelial cells. Neither IEC-6 nor Caco-2 cells exhibited significant cytotoxic or genotoxic responses at concentrations up to 10 mg/mL. ROS measurements further showed that cranberry juice reduces H_2_O_2_-induced oxidative stress by lowering intracellular ROS levels to or below control values. Interestingly, no clear dose–response relationship was observed, suggesting that even lower concentrations of phenolic compounds in cranberries contribute to cytoprotection, while higher concentrations provide no additional benefit or toxicity. Analysis of phenolic composition offers insights into the possible mechanisms of these protective effects. Raw cranberry juice mainly contained anthocyanins and flavonols, as well as hydroxybenzoic and hydroxycinnamic acids. After simulated gastrointestinal digestion, the total phenolic content decreased to 30.78 mg/100 mL, primarily due to anthocyanin breakdown and partial loss of flavonols, but phenolic acids remained relatively stable. This transformation likely generated metabolites that preserved their antioxidant properties, aligning with the observed ROS-lowering effect of juice digestion. Importantly, the protective effect was evident across the entire tested concentration range without a clear dose–response pattern, indicating that both individual phenolic compounds and the total phenolic content contribute to cytoprotection. Phenolic acids, remaining after digestion, may be essential in maintaining cell viability and genome integrity. In addition, flavonols and anthocyanin metabolites could further aid ROS scavenging and prevent oxidative DNA damage. The synergistic action of these compounds probably supports cell viability, prevents genotoxicity, and reduces oxidative stress. Such mechanisms are supported by other studies on individual and combined phenolic compounds [80,81,82,83], as well as comprehensive reviews of the composition, pharmacotherapy, and potential actions of cranberry constituents [10].

## 3. Materials and Methods

### 3.1. Biological Material

#### 3.1.1. Cranberry Pulp and Juice Preparation

Cranberry fruits (*V. macrocarpon*) were obtained from a local retail market in Łódź, Poland (51°46′36″ N 19°27′17″ E). The fresh berries were rinsed with sterile water, gently dried with a sterile paper towel, crushed, and then frozen at –20 °C. After thawing, the cranberry pulp was pressed using a juice extractor (MES3000, Bosch, Warsaw, Poland). The cloudy juice was filtered through a qualitative paper filter (Whatman, Clifton, NJ, USA) and then passed through a sterile 0.45-μm pore-size membrane filter (Merck-Millipore, Darmstadt, Germany).

Table 3 presents the general composition of Polish cranberry juice, as determined in our previous studies [22].

For experiments with cell lines, the cranberry pulp was centrifuged (10,733× *g*, 15 min, RT), and the resulting juice was filtered through syringe filters with a pore size of 0.45 µm. This yielded raw juice with a pH of 2.5. The juice was then digested in vitro according to the standard INFOGEST gastrointestinal digestion method [84]. In brief, the raw juice was first mixed with simulated salivary fluid (SSF) in a 1:1 (*v*/*v*) ratio and incubated for 2 min at 37 °C with shaking (200 rpm). The salivary mixture contained α-amylase (75 U/mL), CaCl_2_ (1 M) and electrolytes and was adjusted to pH 7.0 prior to use. After the oral phase, simulated gastric fluid (SGF) with pepsin (2000 U/mL) was added to the sample (juice + SSF) in a 1:1 (*v*/*v*) ratio. The pH was adjusted to 3.0 with 1 M HCl. The mixture was incubated for 2 h at 37 °C with shaking (200 rpm). Simulated intestinal fluid (SIF) containing pancreatin and bile salts (10 mM) was then added to the gastric contents in a 1:1 (*v*/*v*) ratio. The pH was adjusted to 7.0 using 1 M NaOH. The samples were incubated for 2 h at 37 °C with continuous shaking. After digestion, the samples were centrifuged (10,733× *g*, 15 min, RT) and filtered through syringe filters with a pore size of 0.45 µm to remove enzymes, undigested residues and sediments. The digested juice was diluted fivefold relative to the raw juice to compensate for volume changes during digestion. The pH of the digested juice was 7.0. These juices (raw and digested) served as the initial samples for the experiments on cell lines.

The raw diluted cranberry juice and the juice after digestion were evaluated by spectrophotometry and measurement of absorbance counts versus wavelength. The UV/Vis spectra for raw and digested cranberry juice are shown in the Supplementary Material and Figure S2.

#### 3.1.2. Yeast Cultures

The non-conventional yeasts *Wickerhamomyces anomalus* C1 NCYC D5299 (GenBank LT908480), *Dekkera bruxellensis* C2 NCYC D5300 strain (GenBank LT908481) and *Rhodotorula mucilaginosa* C3 (GenBank LT908482) were isolated from flavoured mineral water in Poland. Polyphasic identification was based on both biochemical and genetic testing [53]. For yeast cell multiplication, commercial wort broth (Merck, Darmstadt, Germany) was used as the basal culture medium. For shake cultures, conventional liquid media were prepared containing either glucose or sucrose as a carbon source and other organic compounds. The minimal media with 2% (*w*/*v*) glucose or 2% (*w*/*v*) saccharose, 0.1% (*w*/*v*) [(NH_4_)_2_SO_4_, 0.3% (*w*/*v*) KH_2_PO_4_, 0.2% (*w*/*v*) MgSO_4_ × 7H_2_O, 0.05% (*w*/*v*) yeast extract] were sterilised at 121 °C. The minimal medium with glucose (2% *w*/*v*) and cranberry juice (10% *v*/*v*) was used after pH correction (pH = 5.0) by 0.1 M NaOH. The commercial protein soft drink (87% skimmed milk, 12% milk protein, skimmed milk powder, vanilla flavour, sucralose, carotene colourant) was used without sterilisation.

The culture media (20 mL) were poured into sterile 25 mL Erlenmeyer flasks, into which sterile glass carriers (microscope slides, Star Frost 76 × 26 mm, Knittel Glass, Braunschweig, Germany) were placed vertically in such a way that half of the carrier was immersed in the medium while the other part remained outside. A glass carrier was chosen as the reference hydrophilic surface [22,53].

#### 3.1.3. Cell Culture Conditions

Caco-2 cells were obtained from Cell Line Services GmbH (Eppelheim, Germany), and IEC-6 cells were sourced from the German Collection of Microorganisms and Cell Cultures (GmbH). Caco-2 cells were maintained as a monolayer in high-glucose DMEM (Merck Life Science, Warsaw, Poland). The IEC-6 cells were grown in a 1:1 (*v*/*v*) mixture of low-glucose DMEM and RPMI 1640 (Merck Life Science, Poland). The media were supplemented with 10% foetal bovine serum (FBS) for Caco-2 and 5% FBS for IEC-6 (Thermo Fisher Scientific, Waltham, MA, USA), GlutaMAX™ at a final concentration of 4 mM (Caco-2) or 2 mM (IEC-6) (Thermo Fisher Scientific), 25 mM HEPES (Merck Life Science, Poland), a standard antibiotic solution (100 µg/mL streptomycin and 100 IU/mL penicillin; Merck Life Science, Poland), and 0.1 U/mL insulin for IEC-6 cells (Merck Life Science, Poland). Both cell lines were cultured at 37 °C in a humidified atmosphere containing 5% CO_2_ (Galaxy 48S incubator, New Brunswick, UK) and subcultured every 7–10 days once they reached approximately 80% confluence. The culture medium was refreshed two to three times per week after rinsing the cells with phosphate-buffered saline (PBS; pH 7.2, Merck Life Science, Poland). For passaging, cells were detached using TrypLE™ Express (Thermo Fisher Scientific) and incubated for 8–10 min at 37 °C. The cell suspension was then centrifuged (307× *g*, 5 min), the supernatant was discarded, and the pellet was resuspended in fresh culture medium. Cell number and viability were assessed using a hemocytometer and the trypan blue exclusion method. Only cultures with viability above 90% were used for further experiments.

Caco-2 and IEC-6 cell lines were selected as complementary in vitro models of intestinal epithelium to evaluate the cytotoxic, genotoxic and antioxidant effects of raw and digested cranberry juices intended for use as natural food preservatives. Caco-2 cells derived from human colon adenocarcinoma spontaneously differentiate into enterocyte-like monolayers with tight junctions and microvilli and are therefore widely used to model the human intestinal barrier and evaluate the biocompatibility of food-derived compounds. IEC-6 cells are derived from the normal rat small intestinal crypt epithelium and represent non-transformed, healthy intestinal cells, making them suitable for assessing potential toxicity to normal intestinal tissue. The combination of both lines enables comparison between human cancer-derived and non-tumour intestinal cells, increasing the relevance of the results for the assessment of the intestinal safety of cranberry-based formulations.

#### 3.1.4. 3-(4,5-Dimethylthiazol-2-yl)-2,5-diphenyltetrazolium Bromide (MTT) Assay

The effect of cranberry raw and digested juices on cell viability was evaluated using the MTT assay. Caco-2 and IEC-6 cells were seeded in 96-well plates at a density of 10,000 cells per well and incubated for 24 h at 37 °C in a humidified atmosphere of 5% CO_2_. Then, the culture medium was removed and the cells were exposed to different concentrations of the raw or digested juices (0.1–10 mg/mL), each tested in quadruplicate. The exposure times were 24, 48 and 72 h. Non-treated cells served as the negative control (100% viability). After incubation with the samples, the medium was gently aspirated and MTT solution (0.5 mg/mL in PBS) was added to each well. The plates were incubated for 3 h at 37 °C. Subsequently, the MTT solution was removed and the formed formazan crystals were dissolved in dimethyl sulfoxide (DMSO) (Merck Life Science, Poland). Absorbance was measured at 550 nm with a reference wavelength of 620 nm using a microplate reader (TriStar^2^ LB 942, Berthold Technologies GmbH & Co. KG, Bad Wildbad, Germany). Cell viability was expressed as a percentage of the untreated control, according to the formula:

Viability (100%) = (OD_sample_/OD_control_) × 100%.

The results are presented as the mean ± standard deviation (SD) of four independent replicates.

#### 3.1.5. Single-Cell Electrophoresis (Comet) Assay

Cells (1 × 10^5^/sample) were placed in Eppendorf tubes with culture medium and cranberry juice samples (final volume 1 mL, 0.1–5 mg/mL). Samples containing only medium served as negative controls. After 60 min of incubation (37 °C), the cells were centrifuged (15 min, 4 °C, 182× *g*), the supernatants were removed, and the pellets were mixed with Low Melting Point agarose (Merck Life Science, Poland, 37 °C). The suspensions were applied onto slides pre-coated with Normal Melting Point agarose, covered with coverslips, set on a heating plate (ZF6 Premiere Slide Warmer, North Rocks, Australia), then cooled on a chilling plate (Cleaver Scientific, Rugby, UK). The slides were lysed in alkaline buffer (2.5 M NaCl, 100 mM EDTA, 10 mM Tris, 1% Triton X-100, pH 10) for 60 min at 4 °C, followed by DNA unwinding in 300 mM NaOH and 1 mM EDTA for 20 min. Electrophoresis was conducted with the CSL-COM20 system (Cleaver Scientific) in the same buffer (pH > 13) for 20 min at 21 V and 29 mA. The slides were then neutralised, dried, and stained with DAPI (1 µg/mL, 60 min, 4 °C). Comets were observed using a Nikon fluorescence microscope (200×) with a Nikon Digital Sight DS-U3 camera (Amstelveen, The Netherlands) and analysed using Lucia Comet v.7.0 (Laboratory Imaging, Prague, Czech Republic). For each sample, 50 cells were evaluated, and DNA damage was expressed as % tail DNA. The results are presented as the mean ± SEM.

#### 3.1.6. Reactive Oxidant Species (ROS) Assay

In a black 96-well plate, 10,000 cells were loaded into each well and incubated for 24 h at 37 °C (95% CO_2_). Next, the medium was changed to a fresh one, and cranberry juices (raw and digested) at the test concentrations (0.2, 0.6, 2.5, 10 mg/mL) were added on the cell monolayers (in 4 replicates). Simultaneously, H_2_O_2_ was added at a final concentration of 200 µM to induce oxidative stress. In this way, the following experimental groups were obtained: cells treated with juices alone, and cells treated with juices in combination with H_2_O_2_. The negative control consisted of untreated cells in culture medium, while the positive control was cells exposed to 200 µM H_2_O_2_. The samples were then incubated for 2 h (37 °C, 95% CO_2_). After that time, the test samples were removed, the cells were double-washed with HBSS (Hanks’ Balanced Salt, Merck Life Science, Poland) solution, and DCFH–DA (20 µM) was added to each well along with culture media without supplements and incubated for 30 min (37 °C, 5% CO_2_) in the dark. The fluorescence was measured (λ_ex_ 490 nm and λ_em_ 530 nm). The average DCF fluorescence was determined as a percentage (%) relative to that of untreated cells, which was assumed to be 100%. Cells were then incubated for 2 h under standard conditions (37 °C, 5% CO_2_). After incubation, the test solutions were removed, and cells were washed twice with HBSS (Hanks’ Balanced Salt Solution; Merck Life Science, Poland). Subsequently, 20 µM 2′,7′-dichlorodihydrofluorescein diacetate (DCFH-DA) in supplement-free culture medium was added to each well and incubated for 30 min at 37 °C in the dark. Fluorescence was measured at λ_ex = 490 nm and λ_em = 530 nm. The mean 2′,7′-dichlorofluorescein (DCF) fluorescence for each group was expressed as a percentage of the signal obtained from untreated control cells, which was set to 100%.

### 3.2. Polyphenol Content Analysis

The analysis of the profile and composition of phenolic compounds in cranberry juice samples, before and after simulated digestion, was performed using ultra-high-performance liquid chromatography with spectrophotometric detection and mass spectrometry. A Dionex UltiMate 3000 UHPLC+ liquid chromatography system (Thermo Fisher Scientific Inc., Waltham, MA, USA) equipped with a diode array detector and an LC Transcend™ TLX-2 system connected to a Q-Exactive quadrupole-orbitrap hybrid mass spectrometer with a heated electrospray ionisation source (HESI-II) (Thermo Scientific, Hudson, NH, USA) was used according to the procedure described by Królak et al. [85], with some modifications. Chromatograms were recorded at 270 nm for hydroxybenzoic acids, at 320 nm for hydroxycinnamic acids, at 365 nm for flavonols, and at 520 nm for anthocyanins. The identification of phenolic compounds was achieved through a comparative analysis of retention times, spectral characteristics, full mass spectra in both negative and positive ionisation modes, and MS/MS fragmentation patterns. Comparison was enabled by analysing pure standards under identical conditions. The quantitative determination of individual phenolic compounds was performed using the external standard method. The results were expressed in milligrams of phenolic compounds per 100 mL of juice sample (mg/100 mL).

### 3.3. Hydrophobicity Analysis

The MATH test was used to determine the yeast cells’ ability to adhere to hydrocarbons, as a measure of their hydrophobicity. Yeast cultures were harvested by centrifugation at 5000 rpm for 5 min at 4 °C, washed twice in phosphate-buffered saline (PBS, Merck, Darmstadt, Germany), and finally resuspended in the same buffer. The cell suspension was adjusted to an absorbance at 540 nm of approximately 1.0 with PBS, then 2 mL of the yeast suspension was added to 0.4 mL of xylene (Merck, Darmstadt, Germany) and vortexed for 60 s. The two phases were allowed to separate for 5 min at 25 °C. The aqueous phase was carefully removed, and the absorbance at A_540_ nm was measured. The decrease in the absorbance of the aqueous phase was taken as a measure of cell surface hydrophobicity (CSH), which was calculated using the following formula:

CSH (%) = [(A_o_ − A_f_)/A_o_] × 100

where A_o_ and A_f_ are the absorbance before and after extraction with xylene, respectively [49].

### 3.4. Adhesion Analysis

Analysis of yeast cell adhesion to the glass surface was performed using luminometry and microscopy. For the luminometric tests, the glass carrier was removed from the culture medium, rinsed with sterile distilled water, and swabbed using free ATP sampling pens (Merck, Darmstadt, Germany). The measurements were reported in relative light units (RLU) using a HY-LiTE 2 luminometer (Merck, Darmstadt, Germany). In microscopic studies, the yeast cells on each carrier were stained with basic fuchsin (0.5%) and observed using an Olympus BX41 light microscope equipped with a DP72 digital camera. The total cell adhesion area in the observation field was evaluated using the UTHSCA Image Tool 3.0 (http://compdent.uthscsa.edu/dig/itdesc.html; accessed on 15 May 2021) [22,53].

### 3.5. Statistical Analysis

The results of the polyphenol analysis were presented as the mean ± SD of three independent experiments. The results of the statistical analysis of CSH and cell adhesion were presented as the mean ± SD of three independent experiments. Since the data did not follow a normal distribution (Shapiro–Wilk test), non-parametric tests were used to analyse the following experiments: cell surface hydrophobicity and cell adhesion under different environments. Differences in the measured parameters were determined using the Kruskal–Wallis test (KW test), followed by a multiple comparisons test (MCT) to identify significant differences between groups. A *p*-value < 0.05 was considered statistically significant. The Shapiro–Wilk, Kruskal–Wallis and multiple comparisons tests were conducted using Statistica^®^ 13.1 (StatSoft, Tulsa, OK, USA).

Statistical analysis of the results obtained from cell line experiments was performed using one-way ANOVA and Student’s *t*-test to determine significant differences between treatment groups. The analyses were conducted in OriginPro 6.1 (Northampton, MA, USA) at a significance level of *p* ≤ 0.05.

## 4. Conclusions

Cranberry juice samples obtained from Polish fruits displayed diverse phenolic profiles, which differed between the raw juice and the juice subjected to in vitro digestion. The phenolic compounds present before digestion were mainly anthocyanins and flavonols. It was observed that this composition could influence the surface properties and adhesive activity of food spoilage yeasts. In the presence of cranberry juice, the hydrophobicity of yeast cell surfaces was reduced by ten to several dozen times, which also led to a significantly lower degree of cell adhesion. However, it is important to emphasise that preliminary in vitro findings need to be verified under real-world conditions. The polyphenol profiles in cranberry juice changed markedly after simulated digestion, with the most notable reduction seen in anthocyanins and flavonols. Nonetheless, even after digestion, cranberry juice continued to be a source of active phenolic acids and their derivatives. The results indicated that cranberry juice was well tolerated by intestinal epithelial cells, showing no significant cytotoxic or genotoxic effects. Additionally, cranberry juice was found to effectively reduce oxidative stress by maintaining or lowering intracellular ROS levels. Studies using intestinal epithelial cell models suggest that gastrointestinal digestion does not adversely impact the safety or effectiveness of cranberry juice under these conditions. Our findings provide new insights into the biological activity of cranberry juice. Consequently, this study highlights new aspects of cranberry’s action in both single-cell and multicellular systems. The results not only confirm cranberry’s potential as a natural, biocompatible ingredient in food and pharmaceutical products but also encourage further research into other functional applications of this valuable fruit.

## Figures and Tables

**Table 1:** Phenolic compound contents in cranberry juices [mg/100 mL].

Phenolic Compound		Fresh Cranberry Pulp	Raw * Juice	Digested ** Juice
IUPAC Name	Synonym			
**Hydroxybenzoic acids and aldehydes**				
3,4,5-trihydroxybenzoic acid	gallic acid	0.58 ± 0.03	0.18 ± 0.00	0.04 ± 0.00
3,4-dihydroxybenzoic acid	protocatechuic acid	0.82 ± 0.04	1.13 ± 0.01	0.93 ± 0.06
3,5-dihydroxybenzoic acid	5-carboxyresorcinol	0.50 ± 0.02	0.82 ± 0.00	3.16 ± 0.21
3,4-dihydroxybenzaldehyde	protocatechualdehyde	0.83 ± 0.04	0.13 ± 0.00	0.12 ± 0.01
4-hydroxybenzoic acid	*p*-salicylic acid	0.16 ± 0.01	0.82 ± 0.02	0.48 ± 0.01
2,5-dihydroxybenzoic acid	gentisic acid	0.73 ± 0.03	0.17 ± 0.04	0.68 ± 0.05
2,4-dihydroxybenzoic acid	4-hydroxysalicylic acid	0.23 ± 0.01	0.35 ± 0.02	0.37 ± 0.03
4-hydroxy-3-methoxybenzoic acid	vanillic acid	0.27 ± 0.01	0.57 ± 0.05	0.30 ± 0.00
3-hydroxybenzoic acid	*m*-salicylic acid	0.39 ± 0.02	4.60 ± 1.25	2.23 ± 0.17
2,3-dihydroxybenzoic acid	*o*-pyrocatechuic acid	2.76 ± 0.13	0.33 ± 0.26	0.25 ± 0.02
4-hydroxy-3,5-dimethoxybenzoic acid	syringic acid	0.85 ± 0.04	0.08 ± 0.01	0.71 ± 0.01
4-hydroxy-3,5-dimethoxybenzaldehyde	syringaldehyde	0.10 ± 0.00	1.46 ± 0.04	1.41 ± 0.02
benzoic acid		nd ***	nd	0.24 ± 0.01
3-(2-hydroxyphenyl)propanoic acid	melilotic acid	nd	nd	0.01 ± 0.01
**Hydroxycinnamic acids**				
(1S,3R,4R,5R)-3-[(E)-3-(3,4-dihydroxyphenyl)prop-2-enoyl]oxy-1,4,5-trihydroxycyclohexane-1-carboxylic acid	3-*O*-caffeoylquinic acid	0.02 ± 0.00	0.07 ± 0.00	0.23 ± 0.03
(1R,3R,4S,5R)-3-[(E)-3-(3,4-dihydroxyphenyl)prop-2-enoyl]oxy-1,4,5-trihydroxycyclohexane-1-carboxylic acid	5-*O*-caffeoylquinic acid	9.75 ± 0.31	10.94 ± 1.09	5.30 ± 0.06
(E)-3-[4-[(2S,3R,4S,5S,6R)-3,4,5-trihydroxy-6-(hydroxymethyl)oxan-2-yl]oxyphenyl]prop-2-enoic acid	*p*-coumaric acid-*O*-hexoside	0.98 ±0.03	0.53 ± 0.01	0.96 ± 0.13
trans-(3R,5R)-4-[(E)-3-(3,4-dihydroxyphenyl)prop-2-enoyl]oxy-1,3,5-trihydroxycyclohexane-1-carboxylic acid	4-*O*-caffeoylquinic acid	0.78 ± 0.02	0.84 ± 0.00	0.26 ± 0.06
(E)-3-[4-[(2S,3R,4S,5S,6R)-3,4,5-trihydroxy-6-(hydroxymethyl)oxan-2-yl]oxyphenyl]prop-2-enoic acid	*p*-coumaric acid	0.92 ± 0.03	1.24 ± 0.00	2.03 ± 0.15
(E)-3-(4-hydroxy-3-methoxyphenyl)prop-2-enoic acid	ferulic acid	0.09 ± 0.00	0.17 ± 0.00	1.06 ± 1.21
3-(4-hydroxy-3,5-dimethoxyphenyl)prop-2-enoic acid	sinapic acid	0.03 ± 0.00	0.05 ± 0.01	0.10 ± 0.00
(1S,3R,4R,5R)-3,4-bis[[(E)-3-(3,4-dihydroxyphenyl)prop-2-enoyl]oxy]-1,5-dihydroxycyclohexane-1-carboxylic acid	3,4-di-*O*-caffeoylquinic acid	0.05 ± 0.00	0.06 ± 0.00	nd
**Flavonols**				
5,7-dihydroxy-3-[(2S,3R,4S,5R,6R)-3,4,5-trihydroxy-6-(hydroxymethyl)oxan-2-yl]oxy-2-(3,4,5-trihydroxyphenyl)chromen-4-one	myricetin 3-*O*-galactoside	1.94 ± 0.09	4.91 ± 0.01	0.90 ± 0.03
5,7-dihydroxy-3-[(2S,3R,4S,5R)-3,4,5-trihydroxyoxan-2-yl]oxy-2-(3,4,5-trihydroxyphenyl)chromen-4-one	myricetin 3-*O*-xyloside	0.17 ± 0.01	0.43 ± 0.00	0.05 ± 0.01
3-[3,4-Dihydroxy-5-(hydroxymethyl)oxolan-2-yl]oxy-5,7-dihydroxy-2-(3,4,5-trihydroxyphenyl)chromen-4-one	myricetin 3-*O*-arabinoside	0.12 ± 0.01	0.33 ± 0.00	0.08 ± 0.00
3-[(2S,3R,4S,5S,6R)-6-[[(2R,3R,4R,5S,6S)-3,5-dihydroxy-6-methyl-4-[(2S,3R,4S,5S,6R)-3,4,5-trihydroxy-6-(hydroxymethyl)oxan-2-yl]oxyoxan-2-yl]oxymethyl]-3,4,5-trihydroxyoxan-2-yl]oxy-2-(3,4-dihydroxyphenyl)-5,7-dihydroxychromen-4-one	quercetin 3-*O*-glucosyl-rhamnosyl-glucoside	0.39 ± 0.02	1.06 ± 0.00	0.21 ± 0.02
2-(3,4-dihydroxyphenyl)-5,7-dihydroxy-3-[(2S,3R,4S,5R,6R)-3,4,5-trihydroxy-6-(hydroxymethyl)oxan-2-yl]oxychromen-4-one	quercetin 3-*O*-galactoside	3.06 ± 0.14	7.38 ± 0.29	5.34 ± 0.07
2-(3,4-dihydroxyphenyl)-5,7-dihydroxy-3-[(2S,3R,4S,5S,6R)-3,4,5-trihydroxy-6-[[(2R,3R,4R,5R,6S)-3,4,5-trihydroxy-6-methyloxan-2-yl]oxymethyl]oxan-2-yl]oxychromen-4-one	quercetin 3-*O*-rutinoside	0.22 ± 0.01	0.33 ± 0.00	0.19 ± 0.01
2-(3,4-dihydroxyphenyl)-5,7-dihydroxy-3-[(2S,3R,4S,5R)-3,4,5-trihydroxyoxan-2-yl]oxychromen-4-one	quercetin 3-*O*-xyloside	0.39 ± 0.02	0.98 ± 0.00	0.47 ± 0.01
[(2R,3R,4S,5R,6S)-6-[2-(3,4-dihydroxyphenyl)-5,7-dihydroxy-4-oxochromen-3-yl]oxy-3,4,5-trihydroxyoxan-2-yl]methyl (E)-3-(4-hydroxyphenyl)prop-2-enoate	quercetin 3-*O*-coumaroylgalactoside	0.17 ± 0.01	0.54 ± 0.00	0.37 ± 0.01
2-(3,4-dihydroxyphenyl)-5,7-dihydroxy-3-[(2S,3R,4S,5S)-3,4,5-trihydroxyoxan-2-yl]oxychromen-4-one	quercetin 3-*O*-arabinopyranoside	0.30 ± 0.01	0.92 ± 0.00	0.67 ± 0.01
3-[(2S,3R,4R,5S)-3,4-dihydroxy-5-(hydroxymethyl)oxolan-2-yl]oxy-2-(3,4-dihydroxyphenyl)-5,7-dihydroxychromen-4-one	quercetin-3-*O*-arabinouranoside	0.27 ± 0.01	1.07 ± 0.02	0.31 ± 0.02
2-(3,4-dihydroxyphenyl)-5,7-dihydroxy-3-[(2S,3R,4R,5R,6S)-3,4,5-trihydroxy-6-methyloxan-2-yl]oxychromen-4-one	quercetin 3-*O*-rhamnoside	0.52 ± 0.02	1.41 ± 0.03	0.90 ± 0.01
3,5,7-trihydroxy-2-(3,4,5-trihydroxyphenyl)chromen-4-one	myricetin	0.37 ± 0.02	1.01 ± 0.04	0.37 ± 0.00
2-(3,4-dihydroxyphenyl)-3,5,7-trihydroxychromen-4-one	quercetin	0.37 ± 0.02	0.94 ± 0.10	0.04 ± 0.00
**Anthocyanins**				
(2S,3R,4S,5R,6R)-2-[2-(3,4-dihydroxyphenyl)-5,7-dihydroxychromenylium-3-yl]oxy-6-(hydroxymethyl)oxane-3,4,5-triol	cyanidin 3-*O*-galactoside	5.89 ± 0.28	5.27 ± 0.16	nd
(2S,3R,4S,5S)-2-[2-(3,4-dihydroxyphenyl)-5,7-dihydroxychromenylium-3-yl]oxyoxane-3,4,5-triol	cyanidin 3-*O*-arabinoside	4.14 ± 0.20	3.88 ± 0.22	nd
(2S,3R,4S,5R,6R)-2-[5,7-dihydroxy-2-(4-hydroxy-3-methoxyphenyl)chromenylium-3-yl]oxy-6-(hydroxymethyl)oxane-3,4,5-triol(	peonidin 3-*O*-galactoside	6.22 ± 0.29	7.43 ± 0.07	nd
3-[(2R,3S,4S,5R)-3,4-dihydroxy-5-(hydroxymethyl)oxolan-2-yl]oxy-2-(4-hydroxy-3-methoxyphenyl)chromenylium-5,7-diol	peonidin 3-*O*-arabinoside	2.95 ± 0.14	4.03 ± 0.13	nd
**Total hydroxybenzoic acids and aldehydes**		**8.22 ± 0.44**	**10.63 ± 1.71**	**10.92 ± 0.61**
**Total hydroxycinnamic acids**		**12.61 ± 0.40**	**13.90 ± 1.11**	**9.95 ± 1.63**
**Total flavonols**		**8.29 ± 0.39**	**21.30 ± 0.51**	**9.90 ± 0.20**
**Total anthocyanins**		**19.20 ± 0.91**	**20.60 ± 0.58**	**nd**
**Total phenolic compounds**		**48.33 ± 2.14**	**66.44 ± 3.92**	**30.78 ± 2.45**

* raw juice obtained from cranberry pulp after centrifugation and filtration; ** digested juice obtained in vitro according to the standard method; *** nd—not detected.

**Table 2:** DNA damage [%] in IEC-6 and Caco-2 cells after exposure to different concentrations of raw and digested cranberry juices in the alkaline comet assay. Fifty cells were analysed for each treatment. Error bars denote the standard error of the mean (S.E.M.). Values with the same letters are not significantly different (ANOVA, *p* ≤ 0.05). Each value has at least one letter; values sharing no letters are significantly different from each other.

Cell Line	Cranberry Juice Dose [mg/mL]							
	Raw				Digested			
	0.1	0.3	1.3	5.0	0.1	0.3	1.3	5.0
IEC-6	2.46 ± 0.33 ^a^	7.19 ± 2.10 ^d^	4.42 ± 0.50 ^b^	3.73 ± 0.58 ^a,b^	4.60 ± 0.86 ^b^	4.72 ± 0.90 ^b^	1.56 ± 0.31 ^a^	6.16 ± 0.99 ^c^
Caco-2	1.98 ± 0.28 ^a^	4.24 ± 1.33 ^b^	4.75 ± 0.82 ^b^	8.72 ± 2.36 ^c^	1.77 ± 0.37 ^a^	2.10 ± 0.30 ^a^	4.07 ± 0.80 ^b^	7.38 ± 1.24 ^c^

**Table 3:** Main components of cranberry pulp [22].

Component		Concentration [g/100 mL]
**Saccharides**	Glucose	2.28 ± 0.53
	Fructose	1.84 ± 0.34
**Organic acids**	Citric acid	2.08 ± 0.41
	Malic acid	0.90 ± 0.15
	Quinic acid	0.14 ± 0.04

## Data Availability

The original contributions presented in this study are included in the article. Further inquiries can be directed to the corresponding author.

## Supplementary Materials

The following supporting information can be downloaded at https://www.mdpi.com/article/10.3390/molecules31030418/s1, Figure S1: Effect of raw (R) and digested (D) cranberry juice (CJ) on the viability of IEC-6 and Caco-2 cells in MTT assay after 24, 48 and 72 h of exposure. Each value represents the mean of four repeats ± the standard deviation (SD). The same letters denote statistical differences between results for CJ-R and CJ-D at the same tested concentration and time point (ANOVA, *p* ≤ 0.05). Figure S2: Absorbance profiles versus wavelength for (A) raw cranberry juice and (B) cranberry juice after digestion. Table S1: Cell surface hydrophobicity (CSH) of yeast strains, namely *Wickerhamomyces anomalus* C1, *Dekkera bruxellensis* C2, and *Rhodotorula mucilaginosa* C3, in different environments: minimal medium with glucose, minimal medium with saccharose, a commercial protein drink, and minimal medium with glucose and 10% (*v*/*v*) cranberry juice. Horizontal comparison of cell hydrophobicity between yeast strains. Table S2: Comparison of cell surface hydrophobicity (CSH) in different media, including minimal medium with glucose, minimal medium with sucrose, commercial protein drink, and minimal medium with glucose and 10% (*v*/*v*) cranberry juice, for each tested yeast strain: *Wickerhamomyces anomalus* C1, *Dekkera bruxellensis* C2, and *Rhodotorula mucilaginosa* C3. Vertical comparison of CSH in different cultivation media. Table S3: Adhesion of yeast cells: *Wickerhamomyces anomalus* C1, *Dekkera bruxellensis* C2, and *Rhodotorula mucilaginosa* C3 in various culture media: minimal medium with glucose, minimal medium with saccharose, commercial protein drink, and minimal medium with glucose and 10% (*v*/*v*) cranberry juice, after 9 days of incubation. Horizontal comparison of adhesion for yeast strains in different culture media. Table S4: Adhesion of yeast cells, namely *Wickerhamomyces anomalus* C1, *Dekkera bruxellensis* C2, and *Rhodotorula mucilaginosa* C3, in various culture media, including minimal medium with glucose, minimal medium with saccharose, commercial protein drink, and minimal medium with glucose and 10% (*v*/*v*) cranberry juice, after 9 days of incubation. Table S5: Adhesion of yeast cells: *Wickerhamomyces anomalus* C1, *Dekkera bruxellensis* C2, and *Rhodotorula mucilaginosa* C3 in minimal medium with glucose/saccharose and minimal medium with glucose and 10% (*v*/*v*) cranberry juice.
