# Supplementary material for: New Insights into Cranberry Bioactivity: Polyphenol Composition, Adhesive Effects Against Food Spoilage Yeasts, and Influence on Intestinal Cells
Source: Molecules. 2026 Jan 26;31(3):418. doi: 10.3390/molecules31030418 (PMC12898502; doi:10.3390/molecules31030418)
Supplement: Supplementary file 1 [file molecules-31-00418-s001.zip › molecules-4084975-supplementary.pdf]

Supplementary Material

Table S1. Cell surface hydrophobicity (CSH) of yeast strains: *Wickerhamomyces anomalus* C1, *Dekkera bruxellensis* C2, and *Rhodotorula mucilaginosa* C3 in different environments: minimal medium with glucose, minimal medium with saccharose, a commercial protein drink, and minimal medium with glucose and 10% (*v/v*) cranberry juice.

The analysis represents a vertical comparison of cell hydrophobicity across yeast strains. Statistical analysis using the Kruskal-Wallis test (KW test), followed by a multiple comparison test (MCT) to indicate significant differences between the groups. Table S1 displays the results of the MCT post hoc analysis. Groups with "*" indicate where statistical significance was observed.

Incubation time [days]	Yeast strain	Minimal medium with glucose	Minimal medium with saccharose	Commercial protein drink	Minimal medium with glucose and cranberry juice
3	**C1**	10.43 ± 0.38	23.0 ± 1.85	14.8 ± 0.56*	0.47 ± 0,15
	**C2**	9.67 ± 0.40*	21.87 ±0.06	61.93 ± 1.02	0.20 ± 0.17*
	**C3**	56.93 ± 3.95*	45.83 ± 1.71	67.83 ± 1.14 *	1.23 ± 0.32*
	p value MCT test	p ≤0.05	p >0.05	p ≤0.05	p ≤0.05
6	**C1**	41.1 ± 0.20*	22.33 ± 0.61	8.87 ± 0.81	2.13 ± 0.32
	**C2**	49.87 ± 1.16*	21.87 ± 1.45	9.23 ± 0.25	0.33 ± 0.15*
	**C3**	48.27 ± 0.32	10.27 ± 0.47	45.53 ± 0.81	3.37 ± 0.50*
	p value MCT test	p ≤0.05	p >0.05	p >0.05	p ≤0.05
9	**C1**	8.67 ± 1.12*	9.73 ± 1.10*	41.13 ± 0.65 *	2.43 ± 0.83
	**C2**	22.97 ± 0.86	12.07 ± 0.38	49.33 ± 0.71	0.63 ± 0.25*
	**C3**	40.17 ± 1.10*	56.30 ± 0.87*	94.53 ± 2.81*	4.53 ± 0.60*
	p value MCT test	p ≤0.05	p ≤0.05	p ≤0.05	p ≤0.05

Table S2. Comparison of cell surface hydrophobicity (CSH) in different media: minimal medium with glucose, minimal medium with sucrose, commercial protein drink, and minimal medium with glucose and 10% (*v/v*) cranberry juice for each tested yeast strain: *Wickerhamomyces anomalus* C1, *Dekkera bruxellensis* C2, and *Rhodotorula mucilaginosa* C3.

The analysis presents a horizontal comparison of CSH for each analyzed strain in different cultivation media. Statistical analysis was conducted using the Kruskal-Wallis test (KW test), followed by a multiple comparison test (p ≤0.05) to indicate significant differences between the groups. Table S2 displays the results of the MCT post hoc analysis. Groups with "*" indicate where statistical significance was observed.

Incubation time [days]	Yeast strain	Minimal medium with glucose	Minimal medium with saccharose	Commercial protein drink	Minimal medium with glucose and cranberry juice	*p* value MCT test
3	**C1**	10.43 ± 0.38	23.0 ± 1.85	14.8 ± 0.56	0.47 ± 0,15*	p ≤0.05
	**C2**	9.67 ± 0.40	21.87 ±0.06	61.93 ± 1.02	0.20 ± 0.17*	p ≤0.05
	**C3**	56.93 ± 3.95	45.83 ± 1.71	67.83 ± 1.14 *	1.23 ± 0.32*	p ≤0.05
6	**C1**	41.1 ± 0.20*	22.33 ± 0.61	8.87 ± 0.81	2.13 ± 0.32*	p ≤0.05
	**C2**	49.87 ± 1.16*	21.87 ± 1.45	9.23 ± 0.25	0.33 ± 0.15*	p ≤0.05
	**C3**	48.27 ± 0.32*	10.27 ± 0.47	45.53 ± 0.81	3.37 ± 0.50*	p ≤0.05
9	**C1**	8.67 ± 1.12*	9.73 ± 1.10*	41.13 ± 0.65*	2.43 ± 0.83*	p ≤0.05
	**C2**	22.97 ± 0.86	12.07 ± 0.38	49.33 ± 0.71*	0.63 ± 0.25*	p ≤0.05
	**C3**	40.17 ± 1.10*	56.30 ± 0.87*	94.53 ± 2.81*	4.53 ± 0.60*	p ≤0.05

Table S3. Adhesion of yeast cells: *Wickerhamomyces anomalus* C1, *Dekkera bruxellensis* C2, and *Rhodotorula mucilaginosa* C3 in various culture media: minimal medium with glucose, minimal medium with saccharose, commercial protein drink, and minimal medium with glucose and 10% (*v*/*v*) cranberry juice, after 9 days of incubation.

The analysis presents a vertical analysis of yeast strain adhesion for each tested culture medium. Statistical analysis was conducted using the Kruskal-Wallis test (KW test), followed by a multiple comparison test ($p \leq 0.05$) to indicate significant differences between the groups. Table S3 displays the results of the MCT post hoc analysis. Groups with "*" indicate where statistical significance was observed.

Yeast strain	Minimal medium with glucose	Minimal medium with saccharose	Commercial protein drink	Minimal medium with glucose and cranberry juice
C1	98.7 ± 0.71	101.2 ± 0.98	112.6 ± 2.91	25.2 ± 2.28
C2	89.8 ± 0.85 *	75.0 ± 1.83*	101.2 ± 4.54 *	17.0 ± 1.68 *
C3	112.8 ± 3.20*	120.1 ± 3.79 *	167.8 ± 2.51*	46.9 ± 1.79 *
p value MCT test	p ≤0.05	p ≤0.05	p ≤0.05	p ≤0.05

Table S4. Adhesion of yeast cells: *Wickerhamomyces anomalus* C1, *Dekkera bruxellensis* C2, and *Rhodotorula mucilaginosa* C3 in various culture media: minimal medium with glucose, minimal medium with saccharose, commercial protein drink, and minimal medium with glucose and 10% (*v*/*v*) cranberry juice, after 9 days of incubation.

The analysis presents a horizontal comparison of each analyzed strain across different cultivation media. Statistical analysis was conducted using the Kruskal-Wallis test (KW test), followed by a multiple comparison test ($p \leq 0.05$) to indicate significant differences between the groups. Table S4 displays the results of the MCT post hoc analysis. Groups with "*" indicate where statistical significance was observed.

Yeast strain	Minimal medium with glucose	Minimal medium with saccharose	Commercial protein drink	Minimal medium with glucose and cranberry juice	*p* value MCT test
C1	98.7 ± 0.71	101.2 ± 0.98	112.6 ± 2.91*	25.2 ± 2.28*	p ≤0.05
C2	89.8 ± 0.85	75.0 ± 1.83*	101.2 ± 4.54*	17.0 ± 1.68*	p ≤0.05
C3	112.8 ± 3.20	120.1 ± 3.79	167.8 ± 2.51	46.9 ± 1.79	p> 0.05

Since the graph in the main text (Figure 2) shows differences in cell adhesion in the presence of cranberry juice, a simple statistical analysis was performed comparing adhesion in the minimal medium with glucose/saccharose and in the minimal medium with glucose and cranberry juice. The analysis revealed statistically significant differences in adhesion in these culture media.

Table S5. Adhesion of yeast cells: *Wickerhamomyces anomalus* C1, *Dekkera bruxellensis* C2, and *Rhodotorula mucilaginosa* C3 in: minimal medium with glucose/saccharose, and minimal medium with glucose and 10% (*v*/*v*) cranberry juice.

The analysis represents a horizontal comparison of yeast adhesion in two types of medium**.** Statistical analysis was conducted using the U-Mann-Whitney test (UWM test).

Yeast strain	Minimal medium with glucose	Minimal medium with glucose and cranberry juice	*p* value UMW test
C1	98.8 ± 1.46	25.2 ± 2.28	p ≤0.05
C2	89,8 ± 8.21	17.0 ± 1.68	p ≤0.05
C3	112.8 ± 5.08	46.9 ± 1.79	p ≤0.05

The Box and Wisher graph represents results of the U-Mann-Whitney analysis of the Adhesion of yeast cells in minimal medium with glucose/saccharose, *vs* minimal medium with glucose and 10% (v/v) cranberry juice

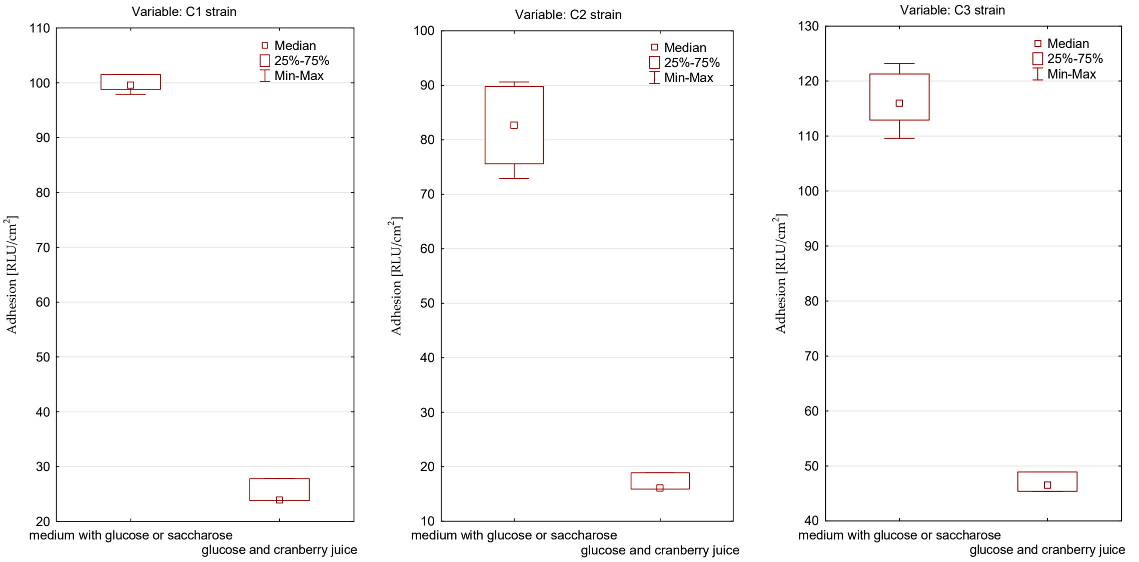

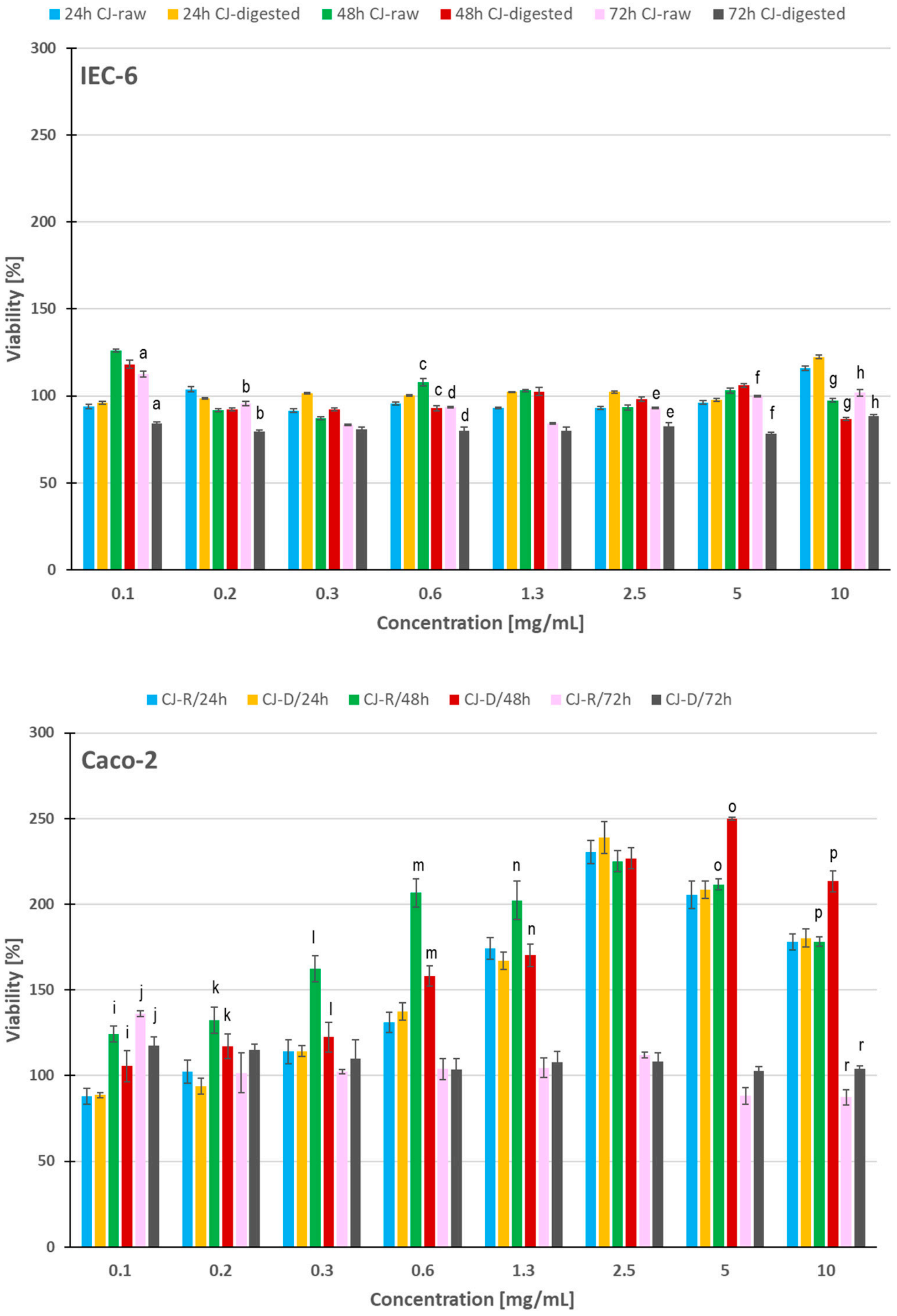

Figure S1. Effect of raw (R) and digested (D) cranberry juice (CJ) on the viability of IEC-6 and Caco-2 cells in MTT assay after 24, 48 and 72 h exposure. Each value represents the mean of four repeats ± the standard deviation (SD). The same letters denote statistical differences between results for CJ-R and CJ-D at the same tested concentration and time point (ANOVA, $p \leq 0.05$).

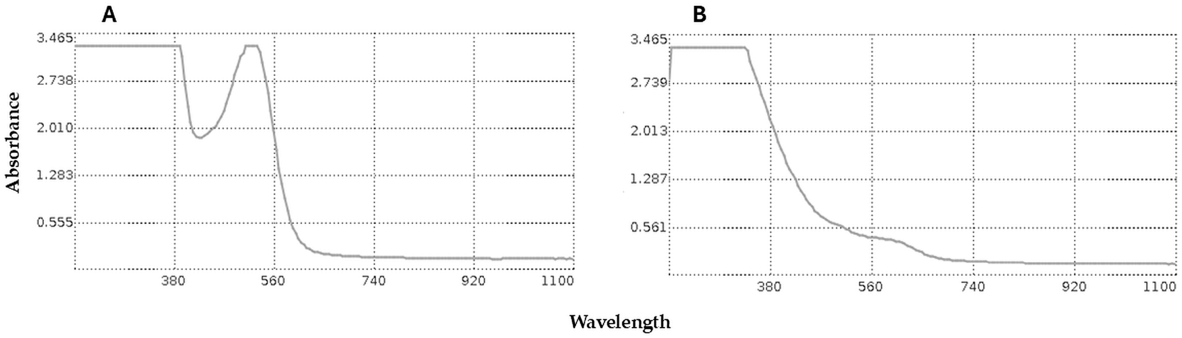

Figure S2. Absorbance profiles versus wavelength for : (A) raw cranberry juice, (B) cranberry juice after digestion.

The profile of cranberry juice show characteristic absorption peak at approximately 525 nm. This wavelength corresponds to the red color of the juice, as the anthocyanin compounds absorb light in the blue, green, and yellow regions of the spectrum. For the cranberry juice after digestion such a characteristic peak was not observed.
